# Oscillometric blood pressure measurement: modeling and analysis of the area oscillogram and height oscillogram

**DOI:** 10.3389/fphys.2025.1611096

**Published:** 2025-06-18

**Authors:** Vishaal Dhamotharan, Hao-Min Cheng, Shih-Hsien Sung, Chen-Huan Chen, Cederick Landry, Mark Freithaler, Aman Mahajan, Sanjeev G. Shroff, Jin-Oh Hahn, Ramakrishna Mukkamala

**Affiliations:** ^1^ Department of Bioengineering, University of Pittsburgh, Pittsburgh, PA, United States; ^2^ School of Medicine, National Yang Ming Chiao Tung University, Taipei, Taiwan; ^3^ Department of Mechanical Engineering, University of Sherbrooke, Sherbrooke, QC, Canada; ^4^ Department of Anesthesiology and Perioperative Medicine, University of Pittsburgh, Pittsburgh, PA, United States; ^5^ Department of Mechanical Engineering, University of Maryland, College Park, MD, United States

**Keywords:** arterial compliance, blood volume oscillations, cuff blood pressure, cuff-arm compliance, mathematical model, oscillometry, parameter estimation, viscoelasticity

## Abstract

**Objective:**

Oscillometry is the most popular blood pressure (BP) measurement method. Conventionally, BP is computed from the oscillation height versus cuff pressure function (“height oscillogram”). However, the oscillation shape also changes with cuff pressure. The objectives were to mathematically model oscillation shape and height variations as a function of cuff pressure and analyze these models using patient data.

**Methods:**

The patient data comprised oscillometric arm cuff pressure and invasive brachial BP waveforms from 109 patients with diverse BPs. The data were analyzed to show that the oscillation area versus cuff pressure function (“area oscillogram”) in particular could be reliably constructed while offering distinct information to the height oscillogram. An analytical model of the area oscillogram was developed with four unknown parameters representing the widths of the brachial artery compliance curve over positive and negative transmural pressure ranges and systolic and diastolic BPs. With invasive systolic and diastolic BPs as inputs, this model and a previous height oscillogram model with the same four parameters, were evaluated in terms of fitting individual patient oscillograms. The impact of key assumptions of the models was evaluated as well.

**Results:**

The area and height oscillogram models fitted the patient data well with errors of 6.9% ± 0.3% and 8.7% ± 0.4%, respectively. Cuff-arm-artery viscoelasticity affected the height oscillogram model fitting, while cuff-arm system nonlinearity may affect area oscillogram model parameter estimates.

**Conclusion:**

Despite simplifying assumptions, the proposed area and previous height oscillogram models can reproduce measured patient oscillograms well. These models may ultimately help improve oscillometric BP measurement accuracy.

## 1 Introduction

Oscillometry has become the preferred non-invasive method for measuring systemic arterial BP, as it is the easiest to use, low in cost, and relatively accurate. Oscillometric arm cuff BP monitors are widely employed in home, office, bedside, and ambulatory settings (Nitzan, 2011). Moreover, oscillometry holds the potential for cuffless BP measurement using everyday devices (Chandrasekhar et al., 2018a; Landry et al., 2024a; Xuan et al., 2023; Panula et al., 2020; Chandrasekhar et al., 2018b).

The oscillometric principle measures BP by exploiting the sigmoidal relationship between blood volume and transmural pressure in arteries, where transmural pressure is the internal BP minus the external pressure. A typical oscillometric device operates by rapidly inflating a cuff around the upper arm to supra-systolic pressures to occlude the underlying brachial artery. The device then deflates the cuff slowly at a rate of 2–4 mmHg/s to a pressure below the diastolic level. As the cuff deflates, the transmural pressure increases, altering the blood volume pulsations. These variable blood volume oscillations proportionally change the volume enclosed by the cuff, thereby inducing oscillations in the cuff pressure. The recorded cuff pressure measurement during the deflation is processed as follows: (i) band-pass filtering to extract the cuff pressure oscillations as a surrogate for the blood volume oscillations and (ii) low-pass filtering to obtain the applied external pressure. These data are then used to compute BP via an algorithm.

Conventional oscillometric algorithms focus on the variable peak-to-peak height of the cuff pressure oscillations relative to the applied cuff pressure (i.e., “height oscillogram”). Popular algorithms that use the height oscillogram to compute BP include the maximum amplitude (Drzewiecki et al., 1994; Mauck et al., 1980; Forouzanfar et al., 2015), fixed ratios (Drzewiecki et al., 1994; Forouzanfar et al., 2015; Geddes et al., 1982), and derivative (Forouzanfar et al., 2015; Drzewiecki and Bronzino, 2006) algorithms. These and other algorithms are population-based or susceptible to noise, leading to significant BP measurement inaccuracies especially beyond normal BP ranges (van Montfrans, 2001; Pickering et al., 2005). However, accurate BP measurement is crucial for reducing the global burden of cardiovascular disease (Mills et al., 2016; Padwal et al., 2019).

**FIGURE 1:**
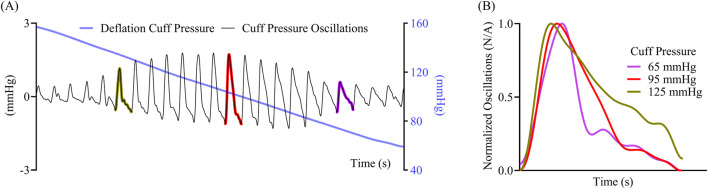
**(A)** Exemplary cuff pressure oscillations and applied cuff pressure measured with an upper arm oscillometric device. **(B)** Variation in the shape of the oscillometric pulses at different cuff pressures.

Figure 1A illustrates an exemplary oscillometric cuff pressure measurement showing variations in the morphology of the oscillometric pulses with decreasing external pressure beyond merely the height variations. The oscillations appear relatively narrow at higher cuff pressures and become wider as the cuff deflates to lower cuff pressures, as shown in Figure 1B. These changes suggest that there may be shape features beyond height that could facilitate the BP computation. We recently analyzed finger oscillometric measurements to show experimentally that analysis of oscillation width variations can yield accurate diastolic BP estimates (Freithaler et al., 2023). Other recent studies have also leveraged shape-based features of individual oscillometric pulses, including oscillation duration, area under the oscillation, and oscillation upstroke and downstroke characteristics, primarily in the context of machine learning-based BP computation (Argha et al., 2019; Celler et al., 2020; Lin et al., 2014).

Mathematical modeling of oscillometry can provide a deeper understanding of the underlying principle and aid in developing more accurate algorithms. Various models, ranging from simple to complex, have been developed (Drzewiecki et al., 1994; Mauck et al., 1980; Chandrasekhar et al., 2019; Raamat et al., 2011; Ursino and Cristalli, 1996; Babbs, 2012; Liu et al., 2016a; Liu et al., 2016b; Forouzanfar et al., 2012). Complex models allow for detailed understanding of all factors that influence the cuff pressure oscillations. However, simple models carry different advantages. We and others previously developed a simple analytical model for the height oscillogram using a parametric sigmoidal function that relates transmural pressure to arterial blood volume (Babbs, 2012; Dhamotharan et al., 2023). We used our parsimonious model to derive simple formulas for readily explaining the three aforementioned algorithms (Chandrasekhar et al., 2019). Furthermore, we and others determined BP and arterial properties by optimally fitting a parsimonious model to the measured height oscillogram, allowing for a patient-specific algorithm (Babbs, 2012; Liu et al., 2016a; Liu et al., 2016b; Forouzanfar et al., 2012; Balasingam et al., 2011). However, to our knowledge, all previous oscillometric modeling efforts have exclusively focused on the height oscillogram.

In this study, we investigated simple shape features of the oscillometric pulses obtained from patient arm cuff pressure measurements. We found that the area under the pulses, when plotted against external pressure, exhibited a consistent inverted U-shape similar to the height oscillogram but with a distinct and easily detectable maximum point. We then developed an analytical model for the “area oscillogram”. We evaluated this model and compared it to our previous height oscillogram model by fitting both models to the patient oscillometric data. Finally, we performed extensive analyses to quantify the impact of key model assumptions on the model fitting. This study may possibly be the first or at least amongst the first to present an analytical model of the area oscillogram or any shape oscillogram for that matter.

## 2 Methods

### 2.1 Patient data

We utilized previously collected high-fidelity data from 128 cardiac catheterization patients for this study. Detailed descriptions of the data and institutional review board (IRB)-approved data collection procedures are available elsewhere (Liu et al., 2016a; Liu et al., 2016b). Briefly, the de-identified patient data comprise single or two consecutive oscillometric cuff pressure waveforms obtained through fast inflation-slow deflation-constant cuff pressure (60 mmHg) cycles of an upper arm cuff device (Watch BP Office, Microlife AG, Switzerland or VP-1000, Omron Colin, Japan). The data include gold standard brachial artery BP waveforms simultaneously measured from the contralateral arm via a micromanometer tipped catheter (SPC-320, Millar Instruments, United States). The measurements were available at baseline conditions and after administration of sublingual nitroglycerin to reduce BP in a subset of the patients. The sampling rate for all waveforms was 250 Hz.

We inspected the data for: (i) inter-arm cuff BP differences of >10 mmHg (Orme et al., 1999), (ii) significant artifact or arrhythmias in the cuff pressure waveforms, (iii) significant brachial BP waveform artifacts, and (iv) oscillograms with incomplete inverted U-shape (>80% amplitude on either side of the maximum) due to insufficient cuff pressure range. After excluding these measurements, a total of 173 waveform pairs from 109 patients remained for analysis. The patient demographics were as follows: 76% male, 61 ± 13 (mean ± SD) years, 163 ± 8 cm, 72 ± 12 kg with arm circumferences of 29 ± 3 cm. The patients had clinical diagnoses of mainly hypertension (61%), coronary artery disease (48%), dyslipidemia (39%) and/or diabetes (24%) and were on various medications. The invasive BP values were 138 ± 20 mmHg for systolic BP, 72 ± 9 mmHg for diastolic BP, and 66 ± 19 mmHg for pulse pressure (PP).

### 2.2 Preliminary analysis to assess shape features of oscillometric pulses

**FIGURE 2:**
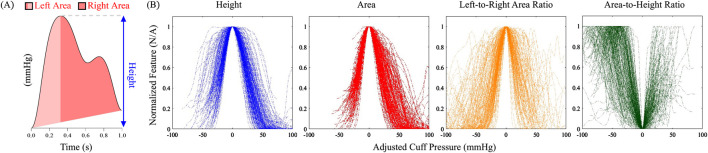
**(A)** Computation of simple shape features of an oscillometric pulse for which analytical modeling is feasible. **(B)** Four features plotted against adjusted cuff pressure for all 173 patient oscillometric measurements in the study. The cuff pressure was adjusted so that the main fiducial marker of all the plots for each feature occurred at 0 mmHg.

We first qualitatively examined four simple features of the oscillometric pulses: (i) oscillation height, (ii) oscillation area, calculated by integrating the pulse amplitudes relative to a line that connects the leading and trailing feet of the pulse over its duration, (iii) oscillation area-to-height ratio, which represents the effective oscillation width, and (iv) ratio of the oscillation areas to the left and right of the systolic peak, which reflects pulse asymmetry. Figure 2A illustrates how these features are computed from an oscillometric pulse. As described below, we extracted clear oscillations from the cuff pressure waveforms; calculated the four features for each oscillation; and plotted them against the applied cuff pressure to generate their respective oscillograms. We aligned each of the oscillograms for the 173 measurements by shifting their fiducial points (maximum for the height, area, and area ratio oscillograms and minimum for the area-to-height ratio oscillogram) to 0 mmHg and superimposed all 173 shifted oscillograms on the same plot, as shown in Figure 2B. Similar to the height oscillogram, the area oscillogram exhibited inverted U-shape.

Both the height and area oscillograms demonstrated consistency across the data with relatively low scatter in the noise-prone low and high cuff pressure ranges, thereby allowing for robust construction. In contrast, the area ratio and area-to-height ratio oscillograms exhibited greater variability across the measurements, indicating that these ratios are more susceptible to signal artifacts and hence may not be reliably formed. Based on the relative quality of the oscillograms, we concluded that the area ratio and area-to-height ratio oscillograms are not ideal for modeling efforts and thus focused on the area oscillogram.

**FIGURE 3:**
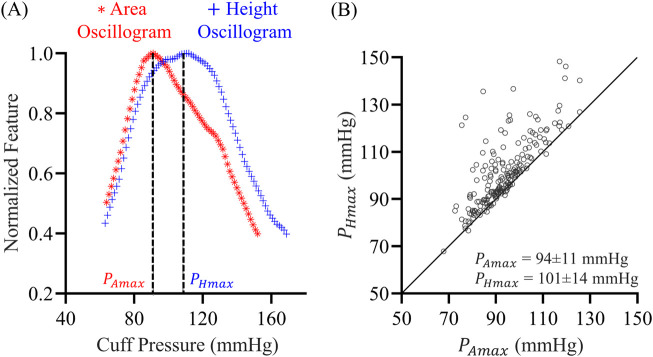
**(A)** Exemplary normalized area oscillogram (oscillation area vs. cuff pressure function) and normalized height oscillogram (oscillation height vs. cuff pressure function) measurements. These oscillograms were constructed from the cuff pressure measurements in Figure 1A. $P_{Amax}$ and $P_{Hmax}$ are the cuff pressures at which the area oscillogram and height oscillogram are maximal, respectively. **(B)** $P_{Hmax}$ plotted against $P_{Amax}$ over the 173 oscillometric measurements.

We compared the area and height oscillograms. As indicated in Figure 3A, the area oscillogram was typically left-shifted relative to the height oscillogram. The maximum amplitudes of the area and height oscillograms occurred at different cuff pressures denoted by $P_{Amax}$ and $P_{Hmax}$ , respectively. When $P_{Hmax}$ was plotted versus $P_{Amax}$ , nearly all the datapoints were above the identity line, as shown in Figure 3B. On average, $P_{Hmax}$ was 7 mmHg higher than $P_{Amax}$ . Additionally, the area oscillogram tended to be narrower than the height oscillogram (see Figure 3A), primarily because of the faster fall with increasing cuff pressure. These observations indicate that the area oscillogram may offer more information about BP and arterial properties to the height oscillogram. Consequently, we proceeded to develop and analyze a mathematical model for the area oscillogram.

### 2.3 Analytical modeling of the area oscillogram

**FIGURE 4:**
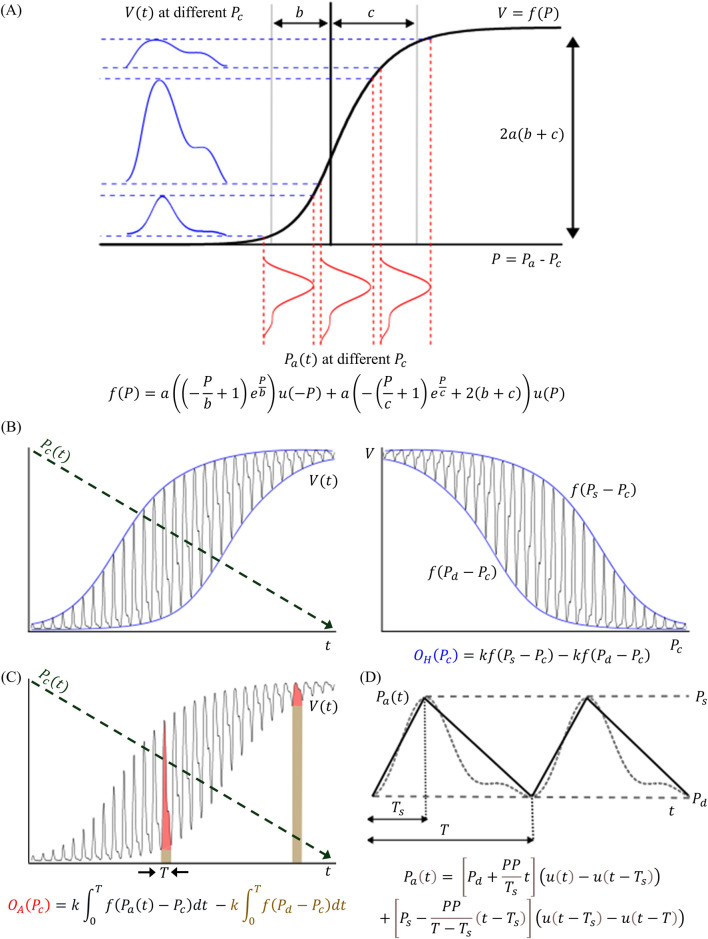
**(A)** The oscillometric models in this study are based on the sigmoidal relationship (*f*(∙)) from transmural pressure ( $P\left(t\right)$ = internal BP ( $\left.P_{a}\left(t\right)\right)$ – external cuff pressure ( $P_{c}$ )) to blood volume ( $V\left(t\right)$ ) in arteries. This relationship is represented by the integral of an exponential-linear function with parameters b and c denoting the widths of the relationship over the negative and positive transmural pressure ranges, respectively, and parameter a determining the height of the relationship (Dhamotharan et al., 2023). $u\left(\cdot \right)$ is the unit step function. **(B)** $V\left(t\right)$ is formed as the response of the model to a slowly decreasing linear cuff pressure ramp $P_{c}$ (t) (left). $V\left(t\right)$ plotted against $P_{c}$ (t) (right) indicates that the previous height oscillogram model ( $O_{H}$ ( $P_{c}$ )) is given by the difference in the x-axis reversed sigmoidal functions evaluated at systolic BP ( $P_{s}$ ) and diastolic BP ( $P_{d}$ ) with scaling by k to convert $V\left(t\right)$ oscillations to the observed cuff pressure oscillations (Chandrasekhar et al., 2019; Dhamotharan et al., 2023). **(C)** The proposed area oscillogram model ( $O_{A}$ ( $P_{c}$ )) is computed as the oscillation area of $kV\left(t\right)$ above the diastolic level for each heartbeat. **(D)** For analytical solution of the area oscillogram integral, $P_{a}\left(t\right)$ is defined as a triangular waveform with parameter PP denoting pulse pressure, parameter T denoting the beat duration, and parameter $T_{s}$ indicating the systolic duration. The dashed line is a real invasive brachial BP waveform for comparison.

Our modeling began with the sigmoidal relationship between transmural pressure ( P ) and blood volume ( V ) in arteries, as depicted in Figure 4A. This relationship is defined by the function $f\left(\cdot \right)$ as follows:

$$V=f\left(P\right)=f\left(P_{a}-P_{c}\right).$$ (1)

Here, $P_{a}$ refers to the BP within the artery or the internal pressure, while $P_{c}$ is the cuff pressure that is assumed to be the external pressure surrounding the artery. By inputting the BP waveform $P_{a}\left(t\right)$ and a slowly decreasing linear cuff pressure ramp $P_{c}\left(t\right)$ into $f\left(P\right)$ , the blood volume waveform ( $\left.V\left(t\right)\right)$ at different P arises, as illustrated in Figure 4B (left). The model then high-pass filters $V\left(t\right)$ to obtain blood volume oscillations and applies a constant scale factor ( k ) to these oscillations to yield the observed cuff pressure oscillations.

To arrive at our previous model of the height oscillogram ( $O_{H}\left(P_{c}\right)$ , i.e., cuff pressure oscillation height versus applied cuff pressure function) (Chandrasekhar et al., 2019; Dhamotharan et al., 2023), $V\left(t\right)$ is plotted against $P_{c}\left(t\right)$ , as shown in Figure 4B (right). It is evident that the upper and lower envelopes of this plot are the x-axis reversed sigmoidal relationships evaluated at systolic and diastolic BPs ( $P_{s}$ and $P_{d}$ ), respectively. The height oscillogram is thus given as follows:

$$O_{H}\left(P_{c}\right)=kf\left(P_{s}-P_{c}\right)-kf\left(P_{d}-P_{c}\right).$$ (2)

The derivative of $f\left(P\right)$ with respect to P or the “arterial compliance curve” ( $g\left(P\right)$ ) is parameterized by an exponential linear-function, which we previously found to be better than seven other functions for height oscillogram modeling (Dhamotharan et al., 2023), as follows:

$$g\left(P\right)=\frac{df\left(P\right)}{dP}=ae^{\frac{P}{b}}\left(-\frac{P}{b}+1\right)\;u\left(-P\right)+ae^{\frac{-P}{c}}\left(\frac{P}{c}+1\right)u\left(P\right)$$ (3)

$$f\left(P\right)=\int g\left(P\right)\;dP=a\left(2b-P\right)e^{\frac{P}{b}}u\left(-P\right)+\left[-a\left(2c+P\right)e^{\frac{-P}{c}}+2a\left(b+c\right)\right]u\left(P\right),$$ (4)

where $u\left(\cdot \right)$ denotes the unit step function; a signifies the maximal arterial compliance at zero transmural pressure; while b and c describe the widths of the arterial compliance curve over the negative and positive transmural ranges, respectively. Substituting Equation 4 into Equation 2 gives the complete model for $O_{H}\left(P_{c}\right)$ as follows:

$$\begin{array}{l} O_{H}\left(P_{c}\right)=d\left(\left(P_{d}-P_{c}+2c\right)e^{-\frac{P_{d}-P_{c}}{c}}-\left(P_{s}-P_{c}+2c\right)e^{-\frac{P_{s}-P_{c}}{c}}\right)\\ \;\times u\left(P_{d}-P_{c}\right)+d\left(2\left(b+c\right)+\left(P_{d}-P_{c}-2b\right)e^{\frac{P_{d}-P_{c}}{b}}-\left(P_{s}-P_{c}+2c\right)e^{-\frac{P_{s}-P_{c}}{c}}\right)\\ \;\times \left(u\left(P_{c}-P_{d}\right)-u\left(P_{c}-P_{s}\right)\right)+d\left(\left(P_{d}-P_{c}-2b\right)e^{\frac{P_{d}-P_{c}}{b}}-\left(P_{s}-P_{c}-2b\right)e^{\frac{P_{s}-P_{c}}{b}}\right)\\ \;\times u\left(P_{c}-P_{s}\right), \end{array}$$ (5)

where d=a·k .

To formulate a new analytical model of the area oscillogram ( $O_{A}\left(P_{c}\right)$ , i.e., cuff pressure oscillation area versus applied cuff pressure function), we integrated $V\left(t\right)$ scaled by k over each beat duration ( T ) and then subtracted the portion of the area below $P_{d}$ , as depicted in Figure 4C and given mathematically as follows:

$$O_{A}\left(P_{c}\right)=\int_{0}^{T}kf\left(P_{a}\left(t\right)-P_{c}\right)dt-\int_{0}^{T}kf\left(P_{d}-P_{c}\right)dt.$$ (6)

The integral defined by Equation 6 with Equation 4 is of the form $\int xe^{x}dx$ , which may be analytically solvable only when x is a linear function. We thus modeled $P_{a}\left(t\right)$ as a triangular pulse for each heartbeat, as shown in Figure 4D and given mathematically as follows:

$$P_{a}\,\left(t\right)=\left[P_{d}+\frac{PP}{T_{s}}\,t\right]\,\left(u\left(t\right)-u\left(t-T_{s}\right)\right)+\left[P_{s}-\frac{PP}{T-T_{s}}\,\left(t-T_{s}\right)\right]\,\left(u\left(t-T_{s}\right)-u\left(t-T\right)\right),$$ (7)

where $PP=P_{s}-P_{d}$ and $T_{s}$ is the systolic duration (i.e., duration over which $P_{a}\left(t\right)$ rises). Substituting Equation 4 and Equation 7 into Equation 6 and solving the resulting integrals gives the complete model for $O_{A}\left(P_{c}\right)$ as follows:

$$\begin{array}{ll} & O_{A}\left(P_{c}\right)=\left(\frac{dcT}{PP}\left[{\left(3c+P_{s}-P_{c}\right)e}^{\left(\frac{-\left(P_{s}-P_{c}\right)}{c}\right)}-{\left(3c+P_{d}-P_{c}\right)e}^{\left(\frac{-\left(P_{d}-P_{c}\right)}{c}\right)}\right]\right.\\ & \;\left.+dT\left(2c{+P}_{d}-P_{c}\right)e^{\left(\frac{-\left(P_{d}-P_{c}\right)}{c}\right)}\right)u\left(P_{d}-P_{c}\right)\\ & \;+\left(\left(\frac{dbT}{PP}\left[3b-\left(3b-\left(P_{d}-P_{c}\right)\right)e^{\left(\frac{\left(P_{d}-P_{c}\right)}{b}\right)}\right]\right)\right.\\ & \;+\left(\frac{dcT}{PP}\left[\left(3c+\left(P_{s}-P_{c}\right)\right)e^{\left(\frac{-\left(P_{s}-P_{c}\right)}{c}\right)}-3c\right]+2d\left(b+c\right)T\frac{P_{s}-P_{c}}{PP}\right.\\ & \;\left.\left.-dT\left(2b-\left(P_{d}-P_{c}\right)\right)e^{\left(\frac{P_{d}-P_{c}}{b}\right)}\right)\right)\left(u\left(P_{c}-P_{d}\right)-u\left(P_{c}-P_{s}\right)\right)\\ & \;+\left(\frac{dbT}{PP}\left[\left(3b-\left(P_{s}-P_{c}\right)\right)e^{\left(\frac{\left(P_{s}-P_{c}\right)}{b}\right)}-\left(3b-\left(P_{d}-P_{c}\right)\right)e^{\left(\frac{\left(P_{d}-P_{c}\right)}{b}\right)}\right]\right.\\ & \;\left.-dT\left(2b-\left(P_{d}-P_{c}\right)\right)e^{\left(\frac{P_{d}-P_{c}}{b}\right)}\right)u\left(P_{c}-P_{s}\right). \end{array}$$ (8)

Note that the parameter $T_{s}$ does not appear in this final expression for $O_{A}\left(P_{c}\right)$ .

Differentiating Equation 5 and Equation 8 with respect to $P_{c}$ and setting the derivatives to zero yield expressions for the cuff pressure at the maximum of the height oscillogram $P_{Hmax}$ (Chandrasekhar et al., 2019) and at the maximum of the area oscillogram $P_{Amax}$ as follows:

$$P_{Hmax}=P_{d}+b\left[\frac{PP}{b+c}\right]$$ (9)

$$\begin{array}{c} \left(2b-P_{d}+P_{Amax}\right)e^{\left(\frac{P_{d}-P_{Amax}}{b}\right)}+\frac{PP}{b}\left(b-P_{d}+P_{Amax}\right)e^{\left(\frac{P_{d}-P_{Amax}}{b}\right)}+\\ \left(2c+P_{s}-P_{Amax}\right)e^{\left(\frac{P_{Amax}-P_{s}}{c}\right)}-2\left(b+c\right)=0. \end{array}$$ (10)

Equation 10 is not analytically solvable for $P_{Amax}$ and is therefore not insightful. We thus employed a simpler exponential function (Dhamotharan et al., 2023) to define the arterial compliance curve $g\left(P\right)$ as follows:

$$g\left(P\right)=\frac{df\left(P\right)}{dP}=\alpha e^{\frac{P}{\beta }}u\left(-P\right)+\alpha e^{\frac{-P}{\gamma }}u\left(P\right),$$ (11)

where α , β , and γ have analogous meanings to a , b , and c , respectively. Using Equation 11 and following similar steps as before, we developed expressions for $P_{Amax}$ and $P_{Hmax}$ as follows:

$$P_{Hmax}=P_{d}+\beta \left[\frac{PP}{\beta +\gamma }\right]$$ (12)

$$\left(\beta +P_{s}-P_{d}\right)e^{\left(\frac{P_{d}-P_{Amax}}{\beta }\right)}+\gamma e^{\left(\frac{P_{Amax}-P_{s}}{\gamma }\right)}-\left(\beta +\gamma \right)=0.$$ (13)

Equation 13 may likewise not be analytically solvable. However, under typical parameter values for α and β (Dhamotharan et al., 2023), the first term is often much larger than the second term such that Equation 13 may be simplified as follows:

$$P_{Amax}=P_{d}+\beta \ln\left[\frac{\beta +PP}{\beta +\gamma }\right].$$ (14)

These final formulas for $P_{Amax}$ and $P_{Hmax}$ can be readily interpreted.

### 2.4 Model evaluation

**FIGURE 5:**
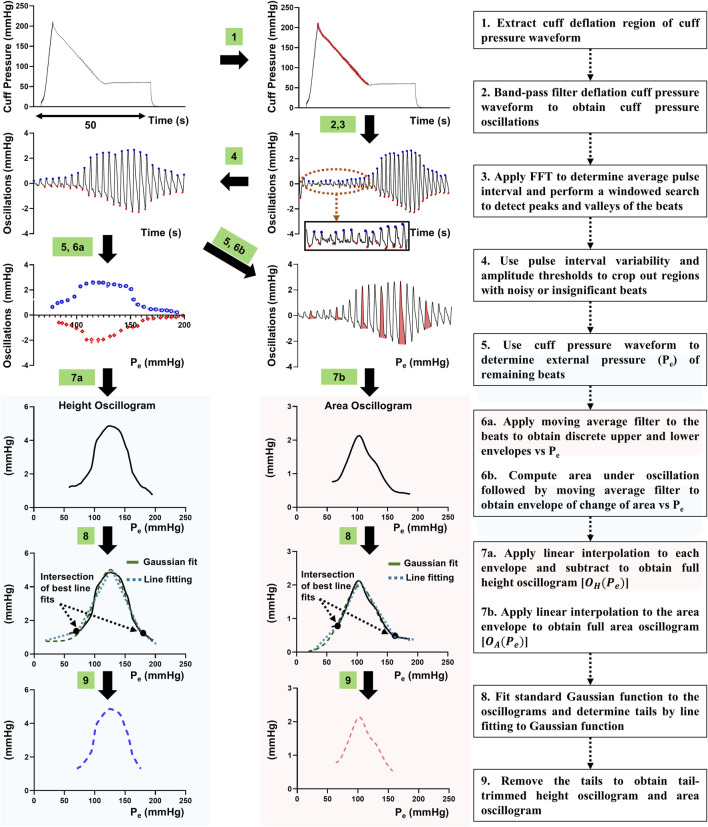
Automated algorithm to form the measured area oscillogram and height oscillogram from the cuff pressure waveform. The cuff pressure waveform, which is measured during fast cuff inflation and then slow cuff deflation followed by a constant cuff pressure of 60 mmHg is analyzed to form tail-trimmed oscillogram measurements. Important user-selected variables: Band-pass filter of 6th order with cut-off frequencies of 0.75 and 5 Hz (step 2); amplitude thresholds *<* 0.2 mmHg for peaks and *> −*0.1 mmHg for valleys and pulse interval variability *<* 0.65/PR and *>* 1.35/PR, where PR is FFT-based pulse rate (steps 3 and 4); and 5^th^ order moving average filter (Step 6).

We evaluated the area and height oscillogram models in terms of their ability to fit the respective patient oscillograms. We constructed the oscillograms from the oscillometric measurements, as shown in Figure 5, using an automated algorithm (Babbs, 2012; Dhamotharan et al., 2023). This algorithm included trimming of the flat tails that can appear at either end of the oscillograms. These tails are not accounted for by our modeling and due to, for example, pulsations from proximal arteries to the cuff or lower pressure vessels under the cuff. We then normalized the trimmed oscillograms by their respective maximum amplitudes. We likewise normalized the oscillogram models of Equations 5, 8. This normalization step eliminated the d parameter in the models. For the $P_{s}$ and $P_{d}$ parameters in the models, we inputted the average systolic and diastolic BPs from the invasive brachial BP waveform over the duration of the oscillogram. We then performed two parameter ( b and c ) quadratic minimizations as follows:

$$\underset{\left\{b,c\right\}}{\mathrm{Min}}\int_{P_{c\_\min}}^{P_{c\_\max}}{\left(O_{x}\left(P_{c}\right)-{\hat{O}}_{x}\left(P_{c},b,c\right)\right)}^{2}dP_{c},$$ (15)

where $O_{x}$ (with $\left.x=A\;or\;H\right)$ represents the measured normalized oscillogram, ${\hat{O}}_{x}$ indicates the normalized oscillogram model fit, and $P_{c\_\min}$ and $P_{c\_\max}$ define the cuff pressure fitting range resulting from the tail trimming. We set the search range for the two parameters b and c to 0–60 mmHg based on our earlier studies (Chandrasekhar et al., 2019; Dhamotharan et al., 2023). We employed sequential quadratic programming to find the minimum over this constrained range using the average b and c parameter values from the previous studies (11 and 14 mmHg) as the initial seeds. For convergence criteria, we set the tolerance for optimality, step, and constraint to 10^−6^. We assessed the performance of the models specifically in terms of the normalized-root-mean-square-error (NRMSE) of the oscillogram fitting in percent as follows:

$$NRMSE=100\cdot \sqrt{\frac{\int_{P_{c\_\min}}^{P_{c\_\max}}{\left(O_{x}\left(P_{c}\right)-{\hat{O}}_{x}\left(P_{c},\hat{b},\hat{c}\right)\right)}^{2}dP_{c}}{\int_{P_{c\_\min}}^{P_{c\_\max}}{\left(O_{x}\left(P_{c}\right)\right)}^{2}dP_{c}}},$$ (16)

where $\hat{b}$ and $\hat{c}$ are the optimal parameter estimates. We also assessed the models by examining the parameter estimates. We used paired t-tests to assess the significance in the difference between the fitting errors and b and c parameter estimates at the p = 0.05 level.

### 2.5 Evaluation of model assumptions

**FIGURE 6:**
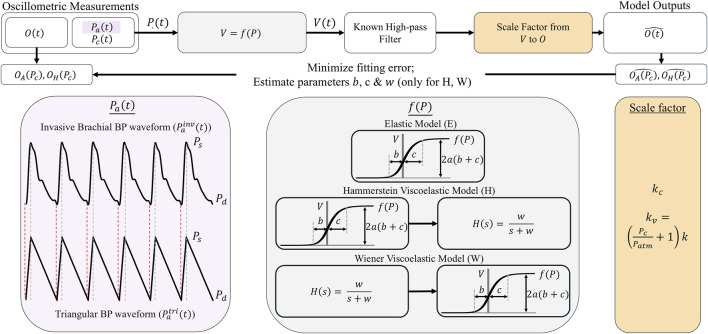
Framework for evaluating the impact of key assumptions of the models on the model fitting errors and parameter estimates. This framework assessed the impact of the triangular BP waveform ( $P_{a}^{tri}\left(t\right)$ ) assumption for the area oscillogram model by comparison with a real simultaneously measured invasive brachial BP waveform ( $P_{a}^{inv}$ (*t*)); the purely elastic model assumption by comparison with standard viscoelastic models with a single additional parameter reflecting the filter cutoff frequency ( w ) (Landry et al., 2024b); and the constant scale factor relating $V\left(t\right)$ oscillations to cuff pressure oscillations ( $O\left(t\right)$ ) by comparison to a model-based variable scale factor where $P_{atm}$ is atmospheric pressure (Drzewiecki et al., 1994).

The oscillogram models of Equations 5, 8 rely on several key underlying assumptions including: (i) a triangular BP pulse for developing the area oscillogram model; (ii) a purely elastic cuff-arm-artery system; and (iii) a constant scale factor to relate blood volume oscillations to cuff pressure oscillations. These assumptions could potentially lead to inaccuracies in the model fits. As shown in Figure 6, we developed a framework to evaluate the impact of these assumptions on the model fitting errors and parameter estimates. The framework essentially involves comparing the fits of the proposed models and alternate models that do not invoke the assumptions to the patient oscillogram measurements.

**FIGURE 7:**
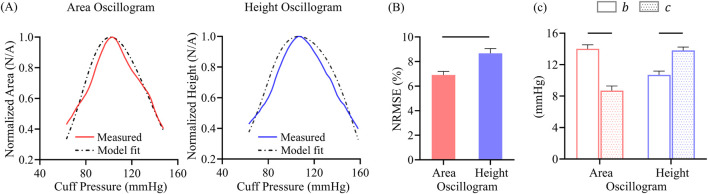
**(A)** Exemplary area oscillogram and height oscillogram model fits with normalized-root-mean-squared-errors (NRMSEs) of 7.5% and 9.1%, respectively. **(B)** NRMSEs and **(C)** parameter estimates of both model fits over all 173 measurements. Data presented as mean ± SE. Horizontal lines indicate significant difference at p < 0.05 level.

**FIGURE 8:**
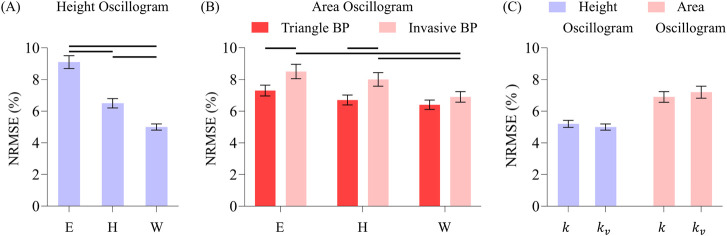
**(A)** Comparison of overall NRMSEs of the height oscillogram model fits for Elastic (E), Hammerstein viscoelastic (H), and Wiener viscoelastic (W) models. **(B)** Comparison of the overall NRMSEs of the area oscillogram model fits for the three models and invasive and triangle BP waveform inputs. Note that the triangle BP waveform input is not assumed by the height oscillogram model. **(C)** Comparison of the overall NRMSEs of both model fits for constant ( k ) and variable ( $k_{v}$ ) scale factors relating blood volume oscillations to cuff pressure oscillations. Although the results were generated using the W model and invasive BP waveform input, they are representative of all comparisons between k and $k_{v}$ scale factors. Data presented as mean ± SE. Horizontal lines indicate significant differences at p < 0.005 level.

To evaluate the triangular BP pulse assumption, we defined two BP waveforms $P_{a}\left(t\right)$ (see purple panels in Figure 6). The first waveform was an alternate invasive brachial BP ( $P_{a}^{inv}\left(t\right)$ ). We applied a high-pass filter ( $f_{c}=0.5$ Hz) to this waveform and re-scaled it using the average $P_{s}$ and $P_{d}$ . The second waveform was the proposed triangular BP ( $P_{a}^{tri}\left(t\right)$ ) generated using Equation 6 but with T and $T_{s}$ determined for each beat based on the invasive BP waveform. This approach ensured a fair comparison, as the two waveforms differed only in the pulse shape. Note that the analytical area oscillogram model of Equation 8 provided comparable fits to using this triangle BP waveform input (compare first bar in Figure 7B to first bar in Figure 8B).

To evaluate the purely elastic cuff-arm-artery system assumption, we defined three systems (see grey panels in Figure 5). The first system was the proposed purely elastic model (E), which included only the static integral of the exponential-linear function $f\left(P\right)$ . The other two systems were alternate Hammerstein (H; static nonlinearity followed by linear damper) and Wiener (W; linear damper followed by static nonlinearity) viscoelastic models, as previously employed for finger arteries in (Landry et al., 2024b). The static nonlinearity in both models was the integral of the exponential-linear function $f\left(P\right)$ in Equation 4, while the linear dynamic component was a first-order, low-pass filter with unity gain and cutoff frequency w (rad/s).

To examine the constant scale factor relating blood volume oscillations to cuff pressure oscillations assumption, we employed a previous physical model of the cuff-arm system (Drzewiecki et al., 1994) in which the nonlinear elasticity of the cuff and compressibility of air within the cuff (Boyle’s law) are incorporated. The model takes the blood volume waveform $V\left(t\right)$ and volume of air pumped into and out of the cuff ( $V_{p}\left(t\right)$ ) as inputs to output the cuff pressure $P_{c}\left(t\right)$ as follows:

$$P_{c}\left(t\right)=E_{C}{\left\{{\left[\frac{\frac{P_{atm}\left(V_{p}\left(t\right)+V_{c0}\right)}{\left(P_{c}\left(t\right)+P_{atm}\right)}+V_{i0}+V\left(t\right)}{V_{i0}+V_{c0}}\right]}^{\frac{1}{n}}-1\right\}}^{n},$$ (17)

where $P_{atm}$ is the atmospheric pressure, $V_{c0}$ is the cuff volume at cuff pressure of 0 mmHg, $V_{i0}$ is the volume of an incompressible arm, and $E_{c}$ and n are parameters defining the nonlinear cuff elasticity. By applying partial derivatives to both sides of Equation 17 at higher $P_{c}$ values, changes in $P_{c}$ are related to changes in V as follows:

$${\partial P}_{c}=\frac{1}{\left[\frac{V_{i0}+V_{c0}}{E_{c}}+\frac{\left(V_{p}+V_{c0}\right)}{P_{atm}{\left(\frac{P_{c}}{P_{atm}}+1\right)}^{2}}\right]}\partial V.$$ (18)

For a standard cuff, $V_{i0}+V_{c0}\ll E_{c}$ and Equation 18 can thus be simplified as follows:

$${\partial P}_{c}=\left(\frac{P_{atm}+P_{c}}{V_{p}+V_{c0}}\right)\left(\frac{P_{c}}{P_{atm}}+1\right)\partial V.$$ (19)

Here, the left term $\left(\frac{P_{atm}+P_{c}}{V_{p}+V_{c0}}\right)$ is the local slope of the $P_{c}-V_{p}$ relationship (i.e., reciprocal of the local cuff-arm compliance) at higher cuff pressures, while the right term $\left(\frac{P_{c}}{P_{atm}}+1\right)$ represents further scaling due to air compression within the cuff induced by arterial pulsations. The patient data used here did not include cuff volume measurements, so we could not study the impact of the nonlinear compliance on the model fitting. We thus could only assess the effect of air compression by arterial expansion and defined two scale factors (see orange panels in Figure 6). The first scale factor was the proposed constant k, and the second scale factor was the alternate variable $k_{v}=\left(\frac{P_{c}}{P_{atm}}+1\right)$ .

Again referring to Figure 6, we fed each of the BP waveforms, $P_{a}^{inv}\left(t\right)$ or $P_{a}^{tri}\left(t\right)$ , along with $P_{c}\left(t\right)$ into each of the three nonlinear models, H, W, or E, to compute $V\left(t\right)$ . We then high-pass filtered this waveform and scaled it by k or $k_{v}$ to compute the cuff pressure oscillations. We constructed the area and height oscillograms using the oscillations. We determined the model parameters by optimal fitting to the patient data. Note that for the viscoelastic models, we employed three parameter ( b,c , and w ) quadratic minimization for the fitting. We evaluated the model fits again in terms of NRMSE and the parameter estimates. We finally invoked paired t-tests to determine differences in the model fitting errors and parameter estimates, using a significance level of p = 0.005 to approximately account for the multiple comparisons involved.

## 3 Results

### 3.1 Formulas for cuff pressure at the oscillogram maximum

The simplified formulas for the cuff pressure at which the height and area oscillograms reach their maximum, $P_{Hmax}$ and $P_{Amax}$ , allow for a qualitative comparison, since they share the same four parameters (see Equations 12, 14). When comparing these two formulas, it is evident that $P_{Amax}$ will consistently be less than $P_{Hmax}$ . This theoretical prediction aligns with the peak positions extracted from the patient oscillogram data (see Figure 3), indicating that the models correctly capture the typical leftward shift of the area oscillogram compared to the height oscillogram.

### 3.2 Oscillogram model fits

Figure 7A shows representative examples of the area oscillogram and height oscillogram model fits with NRMSEs of 7.5% and 9.1% respectively. Figure 7B shows that the area oscillogram and height oscillogram models fit the 173 respective tail-trimmed oscillogram measurements with overall NRMSEs of 6.9% ± 0.3% and 8.7% ± 0.4%. The model fits for the area oscillogram were significantly better than for the height oscillogram. Figure 7C shows the average b and c parameter estimates for the area and height oscillogram model fits. The height oscillogram model fits yielded significantly larger c parameter estimates than b parameter estimates on average, consistent with a right-skewed brachial artery compliance curve (Drzewiecki and Pilla, 1998). However, the parameter estimates from the area oscillogram model fits were unexpected, with the b parameter estimates greater than the c parameter estimates on average.

### 3.3 Effect of assumptions on model fits

Figure 8 shows the overall impact of the different BP waveforms (triangle or invasive) along with the different nonlinear models (Elastic, Hammerstein, or Wiener) and scale factors (constant or variable) on the oscillogram model fitting errors.

For the height oscillogram model fits (see Figure 8A), the NRMSEs were 9.1% ± 0.4%, 6.5% ± 0.4%, and 5.0% ± 0.3% for the Elastic, Hammerstein and Wiener models, respectively. (Note that the triangular BP waveform is not a foundational assumption for the height oscillogram model.) The viscoelastic models yielded significant reductions in the fitting errors by approximately 45% for the Wiener model and about 30% for the Hammerstein model compared to the Elastic model. The substantial fitting error reductions suggest that the additional filter cutoff frequency parameter for the viscoelastic models is crucial for accurately modeling the cuff-arm-artery system response.

For the area oscillogram model fits (see Figure 8B), the NRMSEs for the Elastic, Hammerstein, and Wiener models were 7.3% ± 0.3%, 6.7% ± 0.3%, and 6.4% ± 0.3% for the triangular BP waveform and 8.6% ± 0.5%, 8.0% ± 0.4%, and 6.9% ± 0.3% for the invasive BP waveform. The triangular BP waveform actually yielded significantly better model fitting than the invasive BP waveform for the Elastic and Hammerstein models with an average NRMSE difference of 1.3%. The Wiener model produced significant reductions in NRMSE compared to the Elastic model and the Hammerstein model for the invasive BP waveform. The Wiener model here afforded improvement in the area oscillogram fitting accuracy by 16% on average compared to the Elastic model, which is notably lower than the improvements observed for the height oscillogram model fitting. Interestingly, there were no significant differences in the fitting errors between the Elastic and viscoelastic models for the triangular BP waveform, suggesting that the triangular pulses were not affected by the low-pass filtering effect of the viscoelastic models. These results indicate that the area oscillogram model is more robust to viscoelastic effects than the height oscillogram model.

Finally, the variable scale factor did not have significant impact on the area oscillogram and height oscillogram model fitting errors compared to the constant scale factor, regardless of the BP waveforms or nonlinear models employed. Consequently, only the model fitting errors produced by the Wiener model with the invasive BP waveform input for the two scale factors are shown (see Figure 8C), as these results are representative of the other errors.

**FIGURE 9:**
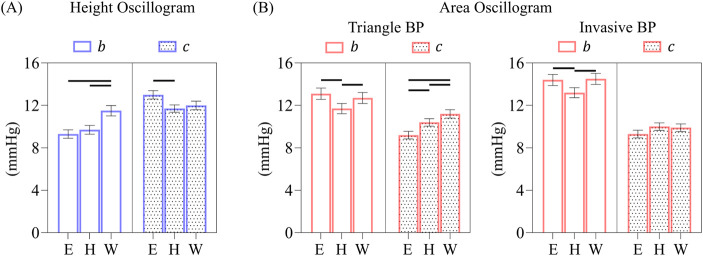
**(A)** Comparison of overall b and c parameter estimates of the height oscillogram model fits for the elastic and two viscoelastic models. **(B)** Comparison of overall b and c parameter estimates of the area oscillogram model fits for the different models and BP waveform inputs. Data presented as mean ± SE. Horizontal lines indicate significant differences at p < 0.005 level.

The b and c parameter estimates for the different BP waveforms and nonlinear models are shown in Figure 9A for the height oscillogram and Figure 9B for the area oscillogram. For the height oscillogram, all models yielded larger c parameter estimates than b parameter estimates in line with known physiological patterns. Compared to the Elastic model, the viscoelastic models altered the b parameter estimates more than the c parameter estimates on average. For the area oscillogram (see Figure 9B), the parameter estimates for c always remained smaller than for b . Compared to the Elastic model, the viscoelastic models impacted the c parameter estimates more than the b parameter estimates on average for the triangular BP waveform. In general, the viscoelastic models brought the b and c parameter estimates closer together, whereas they were significantly different for the Elastic model for both the area and height oscillograms.

The corresponding w parameter estimates were similar for the Hammerstein and Wiener models and for the height and area oscillograms and was 3.1 Hz on average, indicating a significant damping effect. Consistent with the model fitting errors, the variable scale factor did not have significant effect on the parameter estimates.

## 4 Discussion

### 4.1 Area oscillogram

In conventional oscillometry, BP is computed from the cuff pressure oscillation height versus applied cuff pressure function (“height oscillogram”). However, the shape of the oscillometric pulses is also known to change with the cuff pressure (see Figure 1). In this study, we employed an exquisite patient dataset to find that the cuff pressure oscillation area versus applied cuff pressure function (“area oscillogram”) can be robustly measured compared to other shape oscillograms in which analytical modeling is feasible (see Figure 2). Although both the area oscillogram and height oscillogram consistently exhibited inverted-U shape, there were notable differences between the two oscillograms (see Figure 3). With respect to the height oscillogram, the area oscillogram was typically (i) left-shifted (i.e., peaked at lower cuff pressure) and (ii) narrower in width. The oscillation width decreases as the cuff pressure increases, while the oscillation height rises and then falls with increasing cuff pressure. Therefore, the oscillation area increases and decreases more rapidly with increasing cuff pressure than the oscillation height, resulting in a left-shifted and narrower oscillogram.

### 4.2 Parsimonious area oscillogram and height oscillogram models

We then extended our previous work on a parsimonious model for the height oscillogram (Dhamotharan et al., 2023) to develop such a mathematical model for the area oscillogram in this study. Previous modeling efforts may have all exclusively focused on the height oscillogram.

We employed a sigmoid in the form of the integral of an exponential-linear function to relate transmural pressure of an artery to its blood volume and used a constant scale factor to convert blood volume oscillations to the observed cuff pressure oscillations. To obtain a closed-form expression (see Figure 4), we modeled the BP waveform with a triangular pulse parameterized by systolic duration, beat duration, and systolic and diastolic BPs. This approach yielded a model for the area oscillogram, which when normalized, includes four unknown parameters: b and c (negative and positive transmural pressure widths of the arterial compliance curve, which is the derivative of the sigmoidal function) and $P_{s}$ and $P_{d}$ (systolic and diastolic BPs) (see Equation 8). Notably, the systolic duration parameter did not appear in the final expression, whereas the beat duration is measurable. The previous height oscillogram model, which when normalized, includes the same four unknown parameters (see Equation 5).

We also analyzed the models to derive interpretable formulas for the cuff pressure at which the area oscillogram and height oscillogram are maximal using a simpler sigmoid in the form of the integral of an exponential function. These formulas correctly predicted that the peak position of measured area oscillograms typically occurs to the left of the peak position of measured height oscillograms (see Figure 3). However, it is important to note that the area oscillogram model with the exponential-linear function fitted measured area oscillograms with 10% lower NRMSEs than the model with the exponential function on average (result not shown), similar to our earlier findings for the height oscillogram model (Dhamotharan et al., 2023). Therefore, we otherwise used the exponential-linear function for the oscillogram models.

### 4.3 Model fitting results

When we optimally fitted the height oscillogram and area oscillogram models, inputted with invasive brachial systolic and diastolic BPs for $P_{s}$ and $P_{d}$ , to the 173 respective oscillogram measurements (see Figure 5) in the patient dataset, both models provided fits with only <10% error. Furthermore, the new area oscillogram model demonstrated better fitting accuracy than the previous height oscillogram model (see Figures 7A,B), likely because the area or integral of the oscillations is inherently more resilient to measurement noise and pulse irregularities than the height of the oscillations. We thus concluded that both models, and especially the area oscillogram model proposed herein, could fit the data well. It is also worth noting that the area oscillogram and height oscillogram model fitting results were similar for the normotensive subgroup (<140 and <90 mmHg; 51% of patients) and hypertensive subgroup (results not shown). The model fitting results, along with the correct prediction of oscillogram peak positions, indicate that the sigmoidal blood volume-transmural pressure relationship of the artery by itself can account for both height and width changes of the oscillometric pulses.

The b and c parameter estimates obtained via the area oscillogram and height oscillogram model fits were similar in magnitude (8–14 mmHg on average; see Figure 7C). Further, the c parameter estimates increased after sublingual nitroglycerin administration (6.3 ± 3.9 to 8.4 ± 4.5 mmHg for area oscillogram and 12.2 ± 5.0 to 14.2 ± 5.8 mmHg for height oscillogram; results not shown). Such an increase is consistent with the known vasodilatory effect of the drug and suggests the potential clinical value of the parameter estimates. However, an unexpected finding was the contradictory b and c parameter estimate trends from the area oscillogram and height oscillogram model fits (see Figure 7C). The area oscillogram model produced larger b parameter estimates than c parameter estimates. However, the height oscillogram model yielded larger c parameter estimates than b parameter estimates, which aligns with directly measured arterial compliance curve characteristics (Drzewiecki and Pilla, 1998). This latter trend was further demonstrated when we recently applied the height oscillogram model to finger oscillometric measurements (Landry et al., 2024b), confirming b < c for finger arteries as well with a b / c ratio similar to the brachial artery.

The height oscillogram reaches maximal amplitude at a cuff pressure of $P_{Hmax}=\left(\frac{b}{b+c}\right)P_{s}+\left(\frac{c}{b+c}\right)P_{d}$ (see Equation 9). Using the average b and c parameter estimates from the height oscillogram model ( b = 10.7 ± 0.5 and c = 13.8 ± 0.4), $P_{Hmax}=0.43P_{s}+0.57P_{d}$ . This $P_{Hmax}$ formula closely resembles the standard formula used to estimate mean BP (i.e., the time average of the BP waveform) as $0.4P_{s}+0.6P_{d}$ (Bos et al., 2007). This coincidence may explain why $P_{Hmax}$ has been successfully used to compute mean BP (i.e., maximum amplitude algorithm) in traditional oscillometry, although we showed that $P_{Hmax}$ as an estimate of mean BP fails at high PP (Chandrasekhar et al., 2019). In contrast, when using the average parameter estimates from the area oscillogram model ( b = 14.0 ± 0.5, c = 8.7 ± 0.6), we arrive at $P_{Hmax}=0.62P_{s}+0.38P_{d}$ , which would render the maximum amplitude algorithm inaccurate for mean BP estimation across the BP range.

These observations led us to conclude that the parameter estimates from the height oscillogram model were more physiologically representative, while those from the area oscillogram model were compromised to achieve optimal data fitting. We hypothesized that violations to the model assumptions caused this discrepancy in the parameter estimates.

### 4.4 Model assumptions and impact on model fitting

We evaluated the impact of three key model assumptions on the model fitting errors and parameter estimates via a rigorous framework (see Figure 6).

#### 4.4.1 Triangular BP waveform assumption

An obvious error source for the area oscillogram model fits is the assumption of a triangular BP waveform. To assess the impact of this assumption, we compared the fits of the area oscillogram model driven by the real invasive brachial BP waveform and by the presumptive triangular BP waveform (see purple in Figure 6). Interestingly, the model with the triangular BP waveform input yielded a lower area oscillogram model fitting error by 15% on average (see bars over E in Figure 8B). Blood volume oscillations, which manifest as cuff pressure oscillations, are essentially a low-pass filtered version of the BP pulsations due to viscoelastic effects (see below). So, viscoelasticity, which was ignored in this particular analysis, may explain why the smoother triangular BP waveform was able to yield superior fitting over the sharper invasive BP waveform. This analysis also revealed that the input BP waveform type had no impact on the b and c parameter estimates via the area oscillogram model fitting (see bars over E in Figure 9B). The comparative analysis thus justified the triangular BP waveform assumption.

#### 4.4.2 Elastic cuff-arm-artery system assumption

Another major source of model fitting error arises from the assumption that the system comprising the cuff material, arm, and brachial artery is purely elastic. In reality, each of these three components may exhibit viscoelastic behavior across the range of cuff pressures. To assess the impact of this assumption, we compared the fits using the assumed Elastic model (E) and by replacing this model with Wiener (W) or Hammerstein (H) viscoelastic models (see gray in Figure 6).

The Wiener and Hammerstein models afforded significantly more accurate fitting of the measured height oscillograms with error reductions of 45% and 30%, respectively, compared to the Elastic model (see Figure 8A). This finding suggests a significant level of viscoelasticity, as inclusion of just a single parameter $\left(w\right)$ greatly reduced the fitting errors. The impact of viscoelasticity was less pronounced for the area oscillogram model fits (see Figure 8B), as integrating the oscillations to compute their areas effectively acts as a low-pass filter.

Overall, Wiener model provided more accurate fitting of the oscillometric data than both the Hammerstein and Elastic models. Similar results were observed in a previous study on finger oscillometric data (Landry et al., 2024b). Those results were expected, as small finger arteries are rich in smooth muscle and may exhibit a high degree of viscoelasticity. In contrast, brachial arteries are larger with less smooth muscle and should exhibit a lower degree of viscoelasticity. The viscoelastic effects observed in this study may thus also stem from the cuff material and/or arm. The cutoff frequency of the low-pass filter for the viscoelastic models, which indicates the extent of viscoelasticity, was 3.1 Hz on average. This frequency falls within the band-pass filter cutoff frequencies (0.5–5 Hz) used to extract the oscillations from the cuff pressure recordings and is close to typical heart rates (1–2 Hz). These findings highlight why viscoelastic effects cannot be removed by basic signal processing techniques and can significantly impact oscillometric measurements.

The b and c parameter estimates via the two viscoelastic models maintained the trends observed via the Elastic model, with b<c for the height oscillogram model fits and b>c for the area oscillogram model fits (see Figures 9A,B). However, the viscoelastic models produced reductions in the difference in the b and c parameter estimates compared to the Elastic model. These results suggest that viscoelastic effects play a role towards the discrepancy in parameter estimates via the area oscillogram and height oscillogram model fitting but may not fully account for it.

Interestingly, for the height oscillogram model fitting, the viscoelastic models had a more pronounced effect on the b parameter, which primarily affects the higher cuff pressure range. This result suggests viscoelastic effects from the cuff material and artery, as the arm tissue may be fully compressed in the higher cuff pressure range. Similar effects were observed in finger oscillometric measurements, where viscoelasticity led to higher systolic BP estimation errors (Landry et al., 2024b). In contrast, for the area oscillogram model fitting, viscoelasticity had greater influence on the c parameter for the triangular BP waveform input, which mainly impacts the lower cuff pressure range. This result may be due to the viscoelasticity of arm tissue, which begins to decompress at relatively lower cuff pressures. Recall again that the area oscillogram is shifted to the left relative to the height oscillogram and therefore occurs at a lower cuff pressure range.

#### 4.4.3 Constant scale factor relating blood volume to cuff pressure oscillations assumption

A third key assumption of the model is that the blood volume oscillations and cuff pressure oscillations can be related via a constant scale factor. However, the cuff-arm system is known to exhibit significant nonlinearity.

To assess the impact of this assumption, we compared the model fits using the proposed constant scale factor and a variable scale factor of $\left(\left(P_{c}/P_{atm}\right)+1\right)$ (see orange in Figure 6). This variable scale factor accounts for air compression within the cuff due to arterial pulsation, arises from a previous model of the cuff-incompressible arm system (see Equation 19), and can be computed from the cuff pressure and known atmospheric pressure. Although $\left(\left(P_{c}/P_{atm}\right)+1\right)$ linearly increases with $P_{c}$ , the net change (∼5%) over nominal cuff pressure ranges is too small to significantly affect the oscillograms. We accordingly found no significant differences in model fitting errors (see Figure 8C) or in the b and c parameter estimates when using the constant and variable scale factors. This finding confirms our assumption in earlier works that the $\left(\left(P_{c}/P_{atm}\right)+1\right)$ term can be neglected in the scaling from blood volume to cuff pressure oscillations and that the scale factor may thus represent the slope of the cuff pressure-volume of air pumped into and out of the cuff function (i.e., reciprocal of the local cuff-arm compliance; (see Equation 19) (Chandrasekhar et al., 2019; Liu et al., 2016a; Liu et al., 2016b; Dhamotharan et al., 2023).

#### 4.4.4 What assumptions make the model parameters differ between the height oscillogram and area oscillogram?

Collectively, our analysis revealed that none of the studied assumptions significantly impacted the area oscillogram modeling fitting. The analysis further indicated that the assumption of a purely elastic system contributed to the discrepancy in the model parameters from the area oscillogram and height oscillogram modeling fitting. However, is there another assumption that could have caused or contributed to the discrepancy?

**FIGURE 10:**
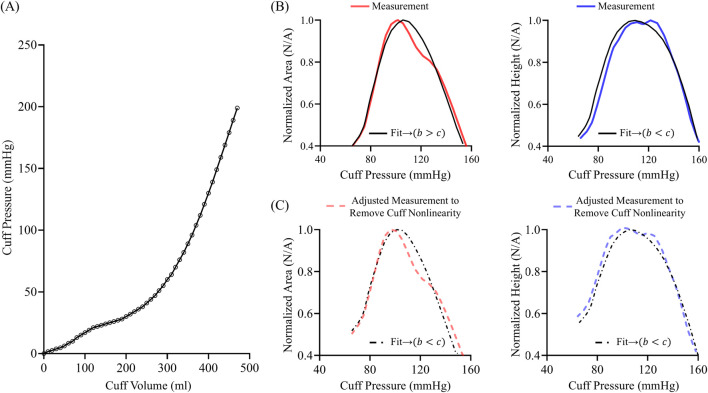
**(A)** Exemplary nonlinear pressure-volume relationship of a standard cuff on a mandrel wrapped with foam to simulate arm tissue. **(B)** Area oscillogram and height oscillogram model fits (black) to measurements (red and blue). **(C)** Area oscillogram and height oscillogram model fits (black) to mathematically adjusted measurements (red and blue) to approximately eliminate cuff nonlinearity in line with **(A)**.

One major error source that we could not rigorously address due to a lack of necessary measurements in the patient dataset is the nonlinear compliance of the cuff-arm system. This nonlinearity is commonly exhibited by standard arm cuffs (Drzewiecki et al., 1994). For example, Figure 10A presents a representative pressure-volume relationship of a universal arm cuff (22–42 cm, Omron BP Monitor, Japan) wrapped around a rigid mandrel with a layer of foam simulating compressible arm tissue. The cuff pressure-volume relationship is highly nonlinear over the low cuff pressure range, becoming approximately linear only at cuff pressures exceeding 100 mmHg. The local slope of the relationship, which is again the reciprocal of the nonlinear cuff-arm compliance and essentially the actual scale factor relating blood volume to cuff pressure oscillations, increased by approximately 150% over the 50–200 mmHg range. Since the nonlinearity is more pronounced at lower cuff pressures, we hypothesized that it affects the area oscillogram more than the height oscillogram and may be responsible for the observed differences in parameter estimates.

We conducted simulations to illustrate the potential impact of cuff-arm compliance nonlinearity on the oscillogram model fitting. We selected a representative oscillometric measurement in which the area oscillogram model fitting yielded b>c parameter estimates, while the height oscillogram model fitting produced the opposite trend. Figure 10B displays the measured area oscillogram and height oscillogram (red and blue) and their respective model fits (black). It is important to note that the oscillogram measurements inherently include the effects of nonlinear compliance from the cuff-arm system. We thus mathematically removed the contribution of the cuff-arm compliance nonlinearity from the oscillogram measurements. First, we varied the scale factor relating blood volume to cuff pressure oscillations linearly from 0.6 to 1 mmHg/mL over the cuff pressure range of 60–100 mmHg and kept it constant above 100 mmHg (in line with Figure 10A). Then, we divided the measured cuff pressure oscillations by this variable scale factor and constructed the area oscillogram and height oscillogram. Figure 10C shows these area oscillogram and height oscillogram measurements adjusted to eliminate the contribution of the cuff-arm compliance nonlinearity (red and blue) along with their respective model fits (black). The adjusted area oscillogram model fit now yielded c>b parameter estimates ( b = 13.5, c = 15.4 vs. b = 15.3, c = 12.1 for analysis that includes nonlinear cuff-arm compliance), while the height oscillogram model fit maintained the original c>b trend for the parameter estimates ( b = 7.8, c = 13.0 vs. b = 8.2, c = 17.0 for analysis that includes nonlinear cuff-arm compliance).

Due to the nature of the nonlinear compliance of the cuff-arm system, the area oscillogram model appreciably adjusted its parameters to achieve the best possible fit. The parameter estimates via the height oscillogram model fit were also impacted but without disrupting the expected trend of b<c . The degree of nonlinearity depends on several factors including the cuff material and sizing, how the cuff is wrapped around the arm, and the arm tissue characteristics. These factors, combined with the BP levels, determine the extent to which the oscillograms are altered compared to a constant scaling. For instance, the nonlinearity may have a higher impact in hospital patients with low BP, obese patients with loose arm tissue but normal BP, and patients with high PP. In sum, nonlinear compliance of the cuff-arm system is a viable explanation for the difference in parameter estimates from the height oscillogram and area oscillogram model fits.

### 4.5 Limitations

Our study has limitations. Firstly, while we rigorously evaluated the new area oscillogram and previous height oscillogram models in terms of how well they explain the respective oscillogram measurements, we have yet to investigate the models in terms of computing BP. Secondly, we ignored arm tissue compression to simplify the modeling. Thirdly, we were not able to rigorously investigate the nonlinear compliance of the cuff-arm system. As explained above, one reason was that necessary cuff volume measurements were not available. Another reason is that modeling the nonlinear compliance would have required adding at least two more parameters to the models, thereby complicating the analysis. Fourthly, while the viscoelastic models used in the study effectively quantified the overall extent of nonlinear dynamics, they did not reveal the individual viscoelastic contributions from the cuff, arm, and artery. Lastly, the findings of this study, based on upper arm cuff measurements, may not be generalizable to oscillometric measurement sites beyond the brachial artery or photoplethysmography measurements of blood volume oscillations.

### 4.6 Implications for oscillometric BP computation

Our study has implications for oscillometric BP computation. The popular fixed ratios algorithm and other conventional oscillometric algorithms only analyze the height oscillogram to compute BP. However, this study indicates that the area oscillogram, which peaks earlier and falls faster than the height oscillogram, offers additional BP information. In particular, the normalized area oscillogram reveals more about the four model parameters (systolic BP, diastolic BP, and the arterial compliance curve widths over negative and positive transmural pressures) than the normalized height oscillogram alone and could therefore potentially help in the BP computation. As a simple example, the peak position of each oscillogram, which may be especially easy to measure, is determined by the four unknown parameters. By analyzing both oscillograms, there would be two equations instead of just one. As a more general example, both models could be optimally fitted to their respective oscillograms, allowing for a patient-specific algorithm that may be more accurate than the conventional population-based algorithms and yield more reliable parameter estimates than patient-specific algorithms that only use the height oscillogram (Liu et al., 2016a; Liu et al., 2016b). Alternatively, the area oscillogram and height oscillogram models could potentially serve as a feature selection guide for machine learning algorithms to improve the BP measurement accuracy. The models may also improve understanding of oscillometric BP computation. For example, our study suggests that cuff-arm-artery system viscoelasticity could adversely impact the computation of systolic BP from the height oscillogram (see Figures 8A, 9A), whereas nonlinear compliance of the cuff-arm system resulting from tissue compression and cuff material may negatively affect the computation of diastolic BP from the area oscillogram (see Figure 10).

## 5 Conclusion

We systematically analyzed extensive and high-fidelity patient data to find that the area oscillogram can be robustly measured and offers complementary information to the conventional height oscillogram about BP and arterial properties. Subsequently, we developed an analytical model of the area oscillogram. We showed that this model fits the patient data well despite its simplifying assumptions. We also provided evidence that the parameter estimates of the area oscillogram model are susceptible to the nonlinear compliance of the cuff-arm system. While the height oscillogram model also provided good fitting to the patient data, we additionally showed here that it was significantly impacted by cuff-arm-artery system viscoelasticity. Our study therefore lays the groundwork for future studies to leverage the oscillogram models to improve oscillometric BP computation. Follow-up work to study the models in the context of tissue compression would also be worthwhile. Ultimately, such subsequent efforts may lead to more accurate oscillometric BP measurement via office, home, and ambulatory (wearable) devices and thereby help improve hypertension control.

## Data Availability

The data analyzed in this study is subject to the following licenses/restrictions: None. Requests to access these datasets should be directed to Chen-Huan Chen (chench@vghtpe.gov.tw).

## References

[B1] Argha A. Wu J. Su S. W. Celler B. G. (2019). Blood pressure estimation from beat-by-beat time-domain features of oscillometric waveforms using deep-neural-network classification models. IEEE Access 7, 113427–113439. 10.1109/access.2019.2933498

[B2] Babbs C. F. (2012). Oscillometric measurement of systolic and diastolic blood pressures validated in a physiologic mathematical model. Biomed. Eng. Online 11, 56. 10.1186/1475-925X-11-56 22913792 PMC3541069

[B3] Balasingam B. Forouzanfar M. Bolic M. Dajani H. Groza V. Rajan S. (2011). “Arterial blood pressure parameter estimation and tracking using particle filters,” in 2011 IEEE international symposium on medical measurements and applications, 473–476.

[B4] Bos W. J. W. Verrij E. Vincent H. H. Westerhof B. E. Parati G. van Montfrans G. A. (2007). How to assess mean blood pressure properly at the brachial artery level. J. Hypertens. 25 (4), 751–755. 10.1097/HJH.0b013e32803fb621 17351365

[B5] Celler B. G. Le P. N. Argha A. Ambikairajah E. (2020). GMM-HMM-Based blood pressure estimation using time-domain features. IEEE Trans. Instrum. Meas. 69 (6), 3631–3641. 10.1109/tim.2019.2937074

[B6] Chandrasekhar A. Kim C. S. Naji M. Natarajan K. Hahn J. O. Mukkamala R. (2018a). Smartphone-based blood pressure monitoring via the oscillometric finger-pressing method. Sci. Transl. Med. 10 (431), eaap8674. 10.1126/scitranslmed.aap8674 29515001 PMC6039119

[B7] Chandrasekhar A. Natarajan K. Yavarimanesh M. Mukkamala R. (2018b). An iPhone application for blood pressure monitoring via the oscillometric finger pressing method. Sci. Rep. 8 (1), 13136. 10.1038/s41598-018-31632-x 30177793 PMC6120863

[B8] Chandrasekhar A. Yavarimanesh M. Hahn J. O. Sung S. H. Chen C. H. Cheng H. M. (2019). Formulas to explain popular oscillometric blood pressure estimation algorithms. Front. Physiol. 10, 10. 10.3389/fphys.2019.01415 31824333 PMC6881246

[B9] Dhamotharan V. Chandrasekhar A. Cheng H. M. Chen C. H. Sung S. H. Landry C. (2023). Mathematical modeling of oscillometric blood pressure measurement: a complete, reduced oscillogram model. IEEE Trans. Biomed. Eng. 70 (2), 715–722. 10.1109/TBME.2022.3201433 36006885 PMC9958264

[B10] Drzewiecki G. (2006). “Noninvasive arterial blood pressure and mechanics,” in The biomedical engineering handbook. Editor Bronzino J. D. 3rd ed. (Boca Raton: CPC Press LLC).

[B11] Drzewiecki G. Hood R. Applet H. (1994). Theory of the oscillometric maximum and the systolic and diastolic detection ratios. Ann. Biomed. Eng. 22, 88–96. 10.1007/BF02368225 8060030

[B12] Drzewiecki G. Pilla J. J. (1998). Noninvasive measurement of the human brachial artery pressure-area relation in collapse and hypertension. Ann. Biomed. Eng. 26 (6), 965–974. 10.1114/1.130 9846935

[B13] Forouzanfar M. Balasingam B. Dajani H. R. Groza V. Z. Bolic M. Rajan S. (2012). “Mathematical modeling and parameter estimation of blood pressure oscillometric waveform,” in 2012 IEEE international symposium on medical measurements and applications proceedings, 1–6.

[B14] Forouzanfar M. Dajani H. R. Groza V. Z. Bolic M. Rajan S. Batkin I. (2015). Oscillometric blood pressure estimation: past, present, and future. IEEE Rev. Biomed. Eng. 8, 44–63. 10.1109/RBME.2015.2434215 25993705

[B15] Freithaler M. Chandrasekhar A. Dhamotharan V. Landry C. Shroff S. G. Mukkamala R. (2023). Smartphone-based blood pressure monitoring via the oscillometric finger pressing method: analysis of oscillation width variations can improve diastolic pressure computation. IEEE Trans. Biomed. Eng. 70 (11), 3052–3063. 10.1109/TBME.2023.3275031 37195838 PMC10640822

[B16] Geddes L. A. Voelz M. Combs C. Reiner D. Babbs C. F. (1982). Characterization of the oscillometric method for measuring indirect blood pressure. Ann. Biomed. Eng. 10 (6), 271–280. 10.1007/BF02367308 7171156

[B17] Landry C. Dhamotharan V. Freithaler M. Hauspurg A. Muldoon M. F. Shroff S. G. (2024a). A smartphone application toward detection of systolic hypertension in underserved populations. Sci. Rep. 14 (1), 15410. 10.1038/s41598-024-65269-w 38965318 PMC11224237

[B18] Landry C. Freithaler M. Dhamotharan V. Daher H. Shroff S. G. Mukkamala R. (2024b). Nonlinear viscoelastic modeling of finger arteries: toward smartphone-based blood pressure monitoring via the oscillometric finger pressing method. IEEE Trans. Biomed. Eng. 71 (9), 2708–2717. 10.1109/TBME.2024.3388316 38625764 PMC11389602

[B19] Lin H. C. Lowe A. Al-Jumaily A. (2014). Non-invasive blood pressure measurement algorithm using neural networks. Artif. Intell. Res. 3 (2). 10.5430/air.v3n2p16

[B21] Liu J. Cheng H. M. Chen C. H. Sung S. H. Moslehpour M. Hahn J. O. (2016a). Patient-specific oscillometric blood pressure measurement. IEEE Trans. Biomed. Eng. 63 (6), 1220–1228. 10.1109/TBME.2015.2491270 26485351 PMC4907878

[B20] Liu J. Cheng H. M. Chen C. H. Sung S. H. Hahn J. O. Mukkamala R. (2016b). Patient-specific oscillometric blood pressure measurement: validation for accuracy and repeatability. IEEE J. Transl. Eng. Health Med. 5 (1900110), 1900110. 10.1109/JTEHM.2016.2639481 29018632 PMC5477767

[B22] Mauck G. W. Smith C. R. Geddes L. A. Bourl J. D. (1980). The meaning of the point of maximum oscillations in cuff pressure in the indirect measurement of blood pressure - Part II. J. Biomech. Eng. 102 (1), 28–33. 10.1115/1.3138195 7382450

[B23] Mills K. T. Bundy J. D. Kelly T. N. Reed J. E. Kearney P. M. Reynolds K. (2016). Global disparities of hypertension prevalence and control: a systematic analysis of population-based studies from 90 countries. Circulation 134 (6), 441–450. 10.1161/CIRCULATIONAHA.115.018912 27502908 PMC4979614

[B24] Nitzan M. (2011). Automatic noninvasive measurement of arterial blood pressure. IEEE Instrum. Meas. Mag. 14 (1), 32–37. 10.1109/mim.2011.5704808

[B25] Orme S. Ralph S. G. Birchall A. Lawson-Matthew P. McLean K. Channer K. S. (1999). The normal range for inter-arm differences in blood pressure. Age Ageing 28 (6), 537–542. 10.1093/ageing/28.6.537 10604505

[B26] Padwal R. Campbell N. R. C. Schutte A. E. Olsen M. H. Delles C. Etyang A. (2019). Optimizing observer performance of clinic blood pressure measurement: a position statement from the lancet commission on hypertension group. J. Hypertens. 37 (9), 1737–1745. 10.1097/HJH.0000000000002112 31034450 PMC6686964

[B27] Panula T. Koivisto T. Pänkäälä M. Niiranen T. Kantola I. Kaisti M. (2020). An instrument for measuring blood pressure and assessing cardiovascular Health from the fingertip. Biosens. Bioelectron. 167, 112483. 10.1016/j.bios.2020.112483 32818750

[B28] Pickering T. G. Hall J. E. Appel L. J. Falkner B. E. Graves J. Hill M. N. (2005). Recommendations for blood pressure measurement in humans and experimental animals: Part 1: blood pressure measurement in humans - a statement for professionals from the subcommittee of professional and public education of the American heart association council on high blood pressure research. Circulation 111 (5), 697–716. 10.1161/01.CIR.0000154900.76284.F6 15699287

[B29] Raamat R. Talts J. Jagomägi K. Kivastik J. (2011). Errors of oscillometric blood pressure measurement as predicted by simulation. Blood Press Monit. 16 (5), 238–245. 10.1097/MBP.0b013e32834af752 21914985

[B30] Ursino M. Cristalli C. (1996). A mathematical study of some biomechanical factors affecting the oscillometric blood pressure measurement. IEEE Trans. Biomed. Eng. 43 (8), 761–778. 10.1109/10.508540 9216149

[B31] van Montfrans G. A. (2001). Oscillometric blood pressure measurement: progress and problems. Blood Press Monit. 6 (6), 287–290. 10.1097/00126097-200112000-00004 12055403

[B32] Xuan Y. Barry C. De Souza J. Wen J. H. Antipa N. Moore A. A. (2023). Ultra-low-cost mechanical smartphone attachment for No-calibration blood pressure measurement. Sci. Rep. 13 (1), 8105. 10.1038/s41598-023-34431-1 37248245 PMC10227087

